# Adolescent Girls and Their Family Members’ Attitudes Around Gendered Power Inequity and Associations with Future Aspirations in Karnataka, India

**DOI:** 10.1177/10778012221097142

**Published:** 2022-08-12

**Authors:** Kalysha Closson, Ravi Prakash, Prakash Javalkar, Tara Beattie, Raghavendra Thalinja, Martine Collumbien, Satyanarayana Ramanaik, Shajy Isac, Charlotte Watts, Stephen Moses, Mitzy Gafos, Lori Heise, Marissa Becker, Parinita Bhattacharjee

**Affiliations:** 1 120479 University of British Columbia, Vancouver, Canada; 2 520510 India Health Action Trust, Lucknow, India; 3 423134 University of Manitoba, Winnipeg, Canada; 4 215824 London School of Hygiene and Tropical Medicine (LSHTM), London, UK; 5 Karnataka Health Promotion Trust (KHPT), Bangalore, India; 6 25802 Johns Hopkins Bloomberg School of Public Health & Johns Hopkins University School of Nursing, Boston, MA, USA

**Keywords:** adolescent girls, education, India, power, gender equity, gender attitudes, future aspirations

## Abstract

Intergenerational differences in inequitable gender attitudes may influence developmental outcomes, including education. In rural Karnataka, India, we examined the extent of intergenerational (adolescent girls [AGs] vs. older generation family members) dis/agreement to attitudes around gendered power inequities, including gender roles and violence against women (VAW). Unadjusted and adjusted logistic regression examined associations between intergenerational dis/agreement to attitude statements and AGs’ future educational and career aspirations. Of 2,457 AGs, 90.9% had a matched family member (55% mothers). While traditional gender roles were promoted intergenerationally, more AGs supported VAW than family members. In adjusted models, discordant promotion of traditional gender roles and concordant disapproval of VAW were associated with greater aspirations. Results highlight the need for family-level programming promoting positive modeling of gender-equitable attitudes.

## Introduction

Globally, pervasive gender inequities, including gender-based violence (GBV), child marriage, and disadvantages in education, have been linked to numerous negative health outcomes and adversely impact global development efforts (World Health Organization [WHO], 2013). The United Nations has set out to achieve specific targets to improve gender equality by eliminating GBV (sustainable development goal [SDG] 5.2) and harmful practices including child marriage (SDG 5.3) (United Nations, 2017).

Gender inequitable and patriarchal attitudes continue to deprive AGs of obtaining quality education and access to adequate sexual and reproductive health and rights (SRHR). Globally, approximately 31 million school-aged girls are not enrolled in school (UNICEF, 2017). Even in school, AGs may not receive the same quality of education as boys and may be subject to harassment and violence both at school and at home (Talboys et al., 2017). These experiences occur at household and community levels, reinforcing unequal gendered power dynamics, normalizing violence against women (VAW), and perpetuating traditional gender roles and harmful gender attitudes (Basu et al., 2017; Blanchard et al., 2018; Zietz & Das, 2017). Previous research has highlighted that across several global contexts, adolescents with parents that hold traditional gender attitudes are more likely to have traditional attitudes themselves (Kagesten et al., 2016). Further, engrained traditional gender attitudes are challenging to shift when held at the household-level. Despite 90% of the global adolescent population living in the global South, most of the research on factors impacting gender attitudes in early adolescence (age 10–14) has been conducted in Western settings (Kagesten et al., 2016). India is home to the largest adolescent population in the world (253 million 10–19-year-olds) (UNICEF, 2020). To inform effective gender-transformative programs, future research is needed in India to examine and describe gender attitudes among adolescents and their family members.

In rural areas, such as the Northern districts of Bijapur and Bagalkot, Karnataka, where there are high rates of poverty, unemployment, and illiteracy, many AGs from lower castes, including schedule caste and schedule tribes (SC/ST) are denied the right to education (Shah, 2011) and are at greater risk of child marriage and early entry into sex work (Prakash et al., 2011, 2017). In 2014, researchers conducted the Samata intervention that aimed to increase secondary school enrollment and reduce HIV risk through, increasing the age of marriage, and entry into the sex trade by working with SC/ST AGs, families, and community members to shift gender norms and attitudes around adolescent gender roles, girls’ education, child marriage, and gender equality (Beattie et al., 2015; Prakash et al., 2017). AGs aged 13–14 enrolled in class 7 at recruitment were included in this study. This age group was selected as it is an important transitional period in which many developmental processes are occurring including physical changes (e.g., onset of menarche) and mental and social development (Prakash et al., 2017). Along with these changes, many young women experience family and community-level restrictions on their mobility, and in turn, often end up being absent from school or dropping out altogether. Furthermore, AGs from SC/ST face numerous socio structural inequities and represent the most disadvantaged groups in India (Kumar, 2007). Previous qualitative research within the Samata intervention has highlighted numerous multilevel barriers to education for AGs from SC/ST including lack of family support, poor quality of education, and negative attitudes around the benefits of educating AGs (Bhagavatheeswaran et al., 2016). Through community-level shifts in gender norms and attitudes, Samata aimed to reduce secondary school drop-out and absenteeism.

The Samata trail data include questionnaire responses from AGs and older generation family members, as such was well positioned to explore differing views on gender attitudes, which have been found to be important drivers of educational attainment (Basu et al., 2017; Beattie et al., 2019; Prakash et al., 2020; Ramanaik et al., 2018; Vyas et al., 2020). Using both data sets (AGs and older generational family members), this study sought to examine intergenerational differences in inequitable, traditional, and harmful gender attitudes toward VAW and gendered power inequities among girls and their older generation family members. In the context of this study, we define gender attitudes as individual perceptions or endorsements of negative gender norms. Although gender attitudes vary widely and have been measured in numerous ways across different global studies (Kagesten et al., 2016), we focus on examining attitudes that are related to gendered power inequities between men and women, including attitudes that endorse traditional gender roles and VAW.

In societies, such as India, where limited mobility of girls is the norm, one could assume that AGs would be likely to hold similar attitudes about gender roles and power dynamics to their parents (Philip et al., 2019). However, little is known about how gender attitudes may differ between AGs and their family members, and in turn what impacts these differences or similarities might have on AGs’ ability to succeed and flourish across the life course. This study aims to explore differences in attitudes toward gendered power inequities among AGs and their older generational family members, as well as associations with AGs’ future aspirations at the baseline of the Samata trial.

## Methods

### Sample and Design

Data for this study were collected from AGs aged 13–14 years and their families from the Vijayapura and Bagalkote districts of northern Karnataka, South India. In these two rural areas, approximately 20% of families are from SC/ST, with many individuals working as seasonal agricultural laborers and most families (>85%) living below the poverty line (Government of Karnataka, Bijapur District, 2008; Office of the Registrar General & Consensus Commissioner, 2011). The data were collected as part of a baseline evaluation of Samata, a cluster-randomized control trial implemented between 2012 and 2017, aiming to reduce the vulnerability to HIV by increasing secondary school completion, increasing the age of marriage, and preventing entry into the sex trade. An equal number of village clusters were randomly assigned to either control (40 village clusters) or intervention (40 village clusters). All 2,457 SC/ST girls aged 13–14 years residing in the 80 village clusters and enrolled in the last year of primary school (standard 7th) were selected to participate in the study in two cohort waves. The girls who consented to participate in the study, along with their parents or older generation family members, were interviewed either between February and April 2014 (Cohort 1) or between September and November 2014 (Cohort 2) as part of the baseline assessment. A detailed sample selection methodology is already described elsewhere (Beattie et al., 2015; Prakash et al., 2017). In brief, all interviews were conducted by female interviewers, lasting around 30–35 min. Interviews with girls and their older generation family members were conducted separately and in private. Interviews were conducted in the local language “Kannada.” In total 2,233 family members of the 2,457 (90.9%) AGs participated in the baseline survey, as such this analysis is restricted to the 2,233 AGs and family pairs included in the baseline survey.

### Exposures of Interest

Understanding the gender attitudes of adolescents is an emerging area of research, and few studies have been conducted in India. A series of questions, used in other studies, was adapted and piloted in this study before commencing the baseline study. To understand similarities and differences in inequitable gender attitudes between AGs and their family members, we examined two questions related to gendered power inequity and VAW that were asked during baseline surveys to both AGs and family members. The two questions of interest have been previously used in other Indian studies examining social norms regarding physical, sexual, and emotional abuse of girls and women (Landry et al., 2020; Vyas et al., 2020). Both answered these questions on a 3-point Likert-type scale ranging from 0–2 (0 = *disagree*, 1 = *somewhat agree*, or 2 = *agree*) to “A wife should always obey her husband” and “There are times when a woman deserves to be beaten.” Because the purpose of the study was to explore the impact of intergenerational differences in attitudes toward gendered power inequities, the main exposure is a measure of the extent to which there is agreement or disagreement between AGs and their family members to these questions (concordant agreement, AGs disagree/family member agree, AGs agree/family member disagree, and concordant disagreement).

### Outcomes of Interest

#### AGs’ Aspirations for the Future

AGs were asked two questions about the level of importance (very, somewhat, and not at all) of (1) completing secondary school and (2) future steady employment.

### Potential Confounders

Socio demographic characteristics were assessed at the individual level for both AGs and their family member as well as household-level. Individual-level characteristics included any male siblings (none vs. ≥1), district (Bagalkot vs. Bijapur), and parental literacy (either/both parent[s] nonliterate [can't read or write] vs. both literate [can read and write]). Household-level characteristics were measured by asking AGs to report the names of all members in the household, and then follow-up questions specified “what is the name of the head of household.” Household-level variables included: sex of the household head (male vs. female), household head literacy (nonliterate [can't read or write] vs. literate [can read and write]), family type (nuclear vs. nonnuclear), standard of living index (low, medium, and high), and self-reported caste (SC vs. ST). The household-level standard of living index was calculated using 13 household asset questions similar to those asked in the national level demographic surveys in India (International Institute for Population Sciences and Macro International, 2007). Principle component analysis was used to generate the weights for generating a composite score.

The relationship of the family member who responded to the questionnaire was grouped as one of the following: mother, father, other male family member, and other female family member. We further included the gender as well as the age of the family member (<35 years, 35–44 years, and 45+ years).

### Ethical Considerations

Ethical approval for the Samata trial was obtained from the Ethical Review Boards of St. John's Medical College (111/2013), the London School of Hygiene and Tropical Medicine (7083), and the University of Manitoba (H2014:414). Parents or legal guardians of the girls provided written informed consent for the girls’ interview, and written assents were taken from the AGs. Independent consent was obtained from the family members for their interview. In those cases where parents or family members were either non-literate or unable to sign the document, witnessed verbal consent was obtained. Data were anonymized by using individual identifiers and participants' names were removed from the AGs’ files.

### Statistical Analysis

Descriptive statistics and frequencies were reported at the individual and household levels. The proportion of responses to both gender attitude questions was assessed among AGs and family members (agree [including agree and somewhat agree] vs. disagree). The level of agreement in responses was assessed descriptively (both agree, AGs disagree but family agree, AGs agree but family disagree, and both disagree). Chi-squared tests examined the bivariate differences between four levels of agreement. After excluding AG/family pairs with missing data, unadjusted and adjusted logistic regression models assessed four levels of agreement to two statements including: (1) *A wife should always obey her husband* and (2) *There are times when a woman deserves to be beaten *and the two outcomes of interest. Adjusted models controlled for household literacy, household composition, family member type, household wealth index, family member age, any male sibling, caste/tribe, and district. Data analysis was conducted on STATA version 13.0 (StataCorp, 2013).

## Results

Overall, 2,230 AGs (aged 13–14) and older generation family members were included in this analysis. The median age of family members was 28 interquartile range = 35–45, and 65.4% were female. The relationship of the family members to the girls was mainly mothers (55.2%), 29.6% of family members were fathers, 11.0% were other male family members, and 4.3% were other female family members. Most family member respondents had no standard education (65.5%), and families were mainly SC (78.5%). At the household level, 78.6% of the household heads were male, 62.7% were illiterate, and just over half consisted of a nuclear family (59.4%) (Table 1).

**Table 1.** Household and Sociodemographic Characteristics of Adolescent Girls (AGs).

	No.	%
*Household-level characteristics*		
Sex of the household head		
Male	1753	78.6
Female	477	21.4
Household head literacy		
Nonliterate	1389	62.7
Literate	828	37.3
Family type		
Nuclear	1325	59.4
Nonnuclear	907	40.6
Standard of living index		
Low	744	33.3
Medium	741	33.2
High	748	33.5
Caste		
Scheduled caste	1752	78.5
Scheduled tribe	481	21.5
*Individual-level characteristics*		
Currently in school		
No	194	8.7
Yes	2039	91.3
District		
Bagalkot	1243	55.7
Bijapur	990	44.3
*Characteristics of family members surveyed*		
Relationship with AG		
Father	660	29.6
Mother	1232	55.2
Other male family member	245	11.0
Other female family member	96	4.3
Parental literacy		
Either/both parent(s) nonliterate	1999	89.6
Both literate	232	10.4
Family member age		
<35 years	541	24.3
35–44 years	1093	49.0
45+ years	496	26.7
Family member sex		
Male	772	34.6
Female	1561	65.4

### A Wife Should Always Obey Her Husband (Statement 1)

The majority of AGs (73.7%) and their family member (93.8%) agreed with this statement. However, among AG–family member pairs, a quarter (24.1%) of AGs disagreed with this statement while their family members agreed, and only 2.3% of pairs both disagreed (Table 2). AGs who agreed (vs. disagreed) to statement 1 were significantly more likely to have a matched family member who also agreed (*p* = .008).

**Table 2.** Extent of Agreement and Disagreement in the Responses of Adolescent Girls (AGs) and Their Family Members on Gender Attitudes.

	AGs		Family members		Extent of agreement between AG and Family				
Gender attitude statements	Agree/somewhat agree (%)	Total *N*	Agree/somewhat agree (%)	Total *N*	Both agree (%)	AG disagree but family agree (%)	AG agree but family disagree (%)	Both disagree (%)	Total *N*
A wife should always obey her husband	73.7	2180	93.8	2227	69.6	24.1	4.0	2.3	2174
There are times when a woman deserves to be beaten	59.0	2208	39.9	2223	23.8	16.2	35.1	24.8	2198

**Figure 1.**
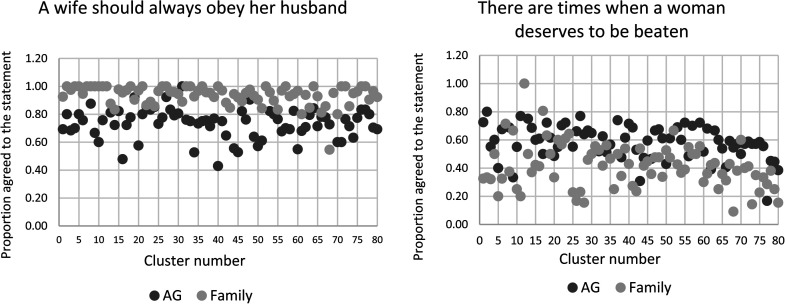
(a) and (b) Proportion of adolescent girl (AG) and family-member agreement to gender attitude statements across clusters.

When examining the level of agreement (0 = both disagree and 1 = both agree) across geographical clusters included in this study, Figure 1(a) shows the relative consistency across clusters, in which family members were more likely to agree with the statement compared to AGs.

Table 3 presents bivariate differences in household-level, individual, and family-member level characteristics across all four agreement levels. Agreement levels within AGs–family-member pairs differed significantly (*p* < .05) between male- versus female-headed households, literate versus nonliterate households, pairs living in Bagalkot versus Bijapur, and across age differences of the family member, where male-headed, nonliterate households in Bagalkot and with older family-member respondents had more inequitable attitudes relating to statement 1.

**Table 3.** Summary Statistics Between Level of Agreement Between Adolescent Girls (AGs) and Their Older Generational Family Member Regarding Gender Roles (*n* = 2,174) and Violence Against Women (*n* = 2,198).

	Summary statistics									
	A wife must obey husband and AG and family-level outcomes (n = 2,174)					There are times when it is ok for a woman to be beaten and AG and family-level outcomes (n = 2,198)				
	Both agree	AG disagree but family agree	AG agree but family disagree	Both disagree	*p*-value	Both agree	AG disagree but family agree	AG agree but family disagree	Both disagree	*p*-value
	*N* (%)	*N* (%)	*N* (%)	*N* (%)		*N* (%)	*N* (%)	*N* (%)	*N* (%)	
	1,514 (69.6)	524 (24.1)	87 (4.0)	49 (2.2)		524 (23.8)	357 (16.2)	772 (35.1)	545 (24.8)	
*Household-level characteristics*										
Sex of the household head										
Male	1,202 (70.5)	398 (23.3)	75 (4.4)	31(1.8)	**.005**	406 (23.5)	277 (16.0)	600 (34.8)	443 (25.7)	.332
Female	309 (66.5)	126 (27.1)	12 (2.6)	18 (3.9)		118 (25.2)	80 (17.1)	170 (36.2)	101 (21.5)	
Household head literacy										
Nonliterate	980 (72.3)	311 (23.0)	40 (3.0)	24(1.8)	**.001**	347 (25.5)	228 (16.7)	484 (35.5)	304 (22.3)	**.006**
Literate	522 (65.0)	209 (26.0)	47 (5.9)	25(3.1)		175 (21.4)	128 (15.6)	282 (24.4)	234(28.6)	
Family type										
Nuclear	910 (70.3)	306 (23.6)	56 (4.3)	23(1.8)	.207	300 (23)	210 (16.1)	451 (34.6)	344 (26.4)	.214
Nonnuclear	604 (68.7)	218 (24.8)	31 (3.5)	26(3.0)		224 (25.1)	147 (16.5)	321 (35.9)	201 (22.5)	
Caste										
Scheduled caste	1170 (68.6)	418 (24.5)	77 (4.5)	41(2.4)	.051	405 (23.5)	280 (16.3)	592 (34.4)	444(25.8)	.193
Scheduled tribe	344 (73.5)	106 (22.6)	10 (2.1)	8(1.7)		119 (24.9)	77 (16.1)	180 (37.7)	101 (21.2)	
Household index					.748					.170
Low	519 (71.0)	170 (23.3)	29 (4.0)	13 (1.8)		172 (23.4)	106 (14.4)	281 (38.3)	175 (23.8)	
Medium	500 (69.7)	175 (24.4)	24 (3.3)	18 (2.5)		189 (26.0)	121 (16.6)	241 (33.1)	176 (34.2)	
High	495 (68.2)	179 (24.7)	34 (4.7)	18 (2.5)		163 (22.1)	130 (17.6)	250 (33.9)	194 (26.3)	
District					**.007**					**.003**
Bagalkot	860 (71.3)	290 (24.0)	39 (3.2)	18 (1.5)		309 (25.0)	204 (16.5)	452 (36.6)	269 (21.8)	
Bijapur	654 (67.6)	234 (24.2)	48 (5.0)	31 (3.2)		215 (22.5)	153 (15.9)	320 (33.2)	276 (28.6)	
Number of male siblings					.255					.612
0	186 (66)	72 (25.5)	14 (5.0)	10 (3.5)		64 (22.5)	51 (18)	105 (37.0)	64 (22.5)	
1+	1328 (70.2)	452 (23.9)	73 (3.9)	39 (2.1)		460 (24)	306 (16)	667 (24.8)	481 (25.1)	
*Characteristics of family members surveyed*										
Relationship of family member to AG					.768					**.036**
Father	442 (69.0)	152 (23.7)	31 (4.8)	16 (2.5)		161 (24.9)	95 (14.7)	221 (34.1)	171 (26.4)	
Mother	836 (69.5)	343 (28.5)	45 (3.7)	24 (2.0)		277 (22.8)	191 (15.7)	438 (36.0)	309 (25.4)	
Other male member	169 (71.9)	60 (25.5)	10 (4.3)	6 (2.6)		54 (22.5)	52 (21.7)	86 (35.8)	48 (20.0)	
Other female member	67 (70.5)	24 (25.3)	<5	<5		32 (33.7)	19 (20.0)	27 (28.4)	17 (17.9)	
Family member sex					.146					.871
Male	521 (69.8)	168 (22.5)	37 (5.0)	21 (2.8)		187 (24.7)	120 (15.9)	260 (34.3)	190 (25.1)	
Female	993 (69.6)	356 (24.9)	50 (3.5)	28 (2.0)		337 (23.4)	237 (16.4)	512 (35.5)	355 (24.6)	
Family member age					**.022**					.123
<35 years	344 (64.4)	138 (26.2)	25 (4.8)	19 (3.6)		146 (27.4)	91 (47.7)	163 (30.6)	132 (24.8)	
35-44 years	764 (71.8)	245 (23.0)	39 (3.7)	16 (1.5)		249 (23.1)	163 (52.2)	399 (37.0)	266 (24.7)	
45+ years	403 (69.4)	141 (24.3)	23 (4.0)	14 (2.4)		128 (21.8)	103 (53.2)	209 (35.7)	146 (24.9)	

**Figure 2.**
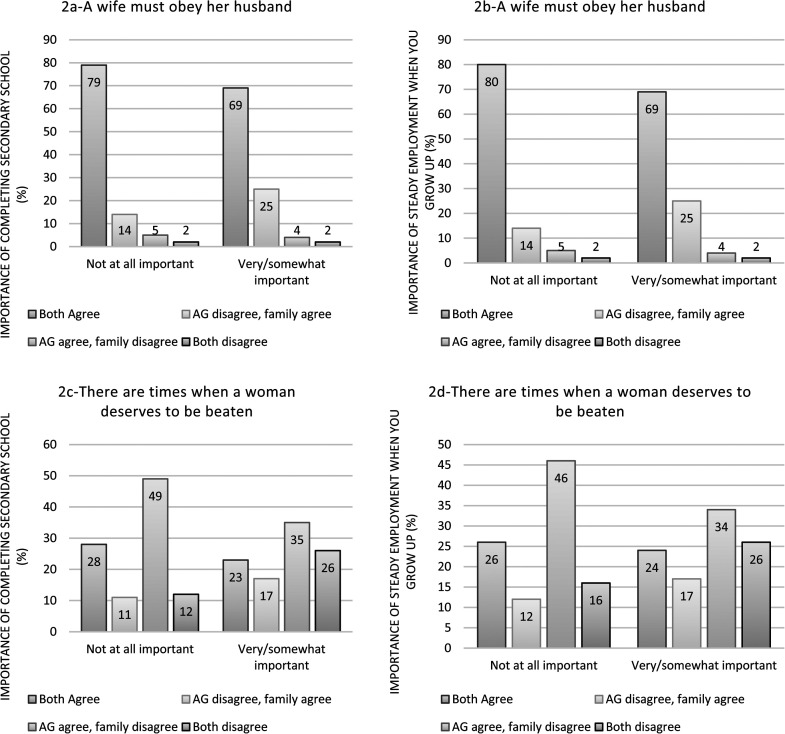
(a)–(d) Proportion of adolescent girls (AGs) reported the importance of completing secondary school and future steady employment by AG–family member pair agreement to gender attitude statements.

When considering gender attitudes against outcomes of interest, Figure 2(a) and (b) shows that a higher proportion of AGs in family pairs in which the AGs disagreed and the family member agreed to statement 1 felt it very/somewhat important to complete secondary school and have steady employment when they grow up. In adjusted models (Table 4) AGs in pairs in which they disagreed and family-member agreed to statement 1 (vs. concordant agreement) were more likely to report that completing secondary school was very/somewhat important (vs. not at all important; adjusted odds ratio [aOR] = 1.99, 95% CI = 1.19–3.34), and that future steady employment was very/somewhat important (vs. not at all important; aOR = 1.95, 95% CI = 1.27–2.98).

**Table 4.** Adjusted Associations Between Statement 1 “A Wife Must Obey Husband” and Samata Girls’ Baseline Aspirations of Educational Attainment and Future Steady Employment.

	Importance of completing secondary school (very/somewhat vs. not at all)	Importance of future steady employment (very/somewhat vs. not at all)
	Adjusted OR (aOR) (95% CI)	aOR (95% CI)
Level of agreement:		
Both agree	Ref	Ref
AG disagrees but family agrees	**1.99 (1.19–3.34)**	**1.95 (1.27–2.98)**
AG agrees but family disagrees	0.56 (0.27–1.18)	0.88 (0.43–1.82)
Both disagree	3.00 (0.40–22.3)	2.45 (0.58–10.26)

*Note*. All models adjusted for household literacy, household composition (e.g., nuclear family), family member type, household wealth index, family member age, any male sibling, caste/tribe, and district.

Items in **bold** represent statistical significance at *p* < .01.

### There Are Times When a Woman Deserves to Be Beaten (Statement 2)

More AGs agreed (59.0%) to this statement compared to their family members (39.9%). Less than a quarter of pairs (24.8%) disagreed with this statement (Table 2). When AGs and family members had discordant responses there were more pairs in which AGs agreed but family disagreed (35.1%) compared to 16.2% of pairs where AGs disagreed, and family members agreed. AGs who agreed (vs. disagreed) to statement 2 were not significantly more likely to have a matched family member who also agreed (*p* = .688). Figure 1(b) shows that across most clusters, AGs were more likely to agree with this statement compared to their family member.

Table 3 presents bivariate differences in household-level, individual, and family-member level characteristics across all four agreement levels. Agreement levels within pairs differed significantly between literate versus nonliterate households, pairs living in Bagalkot versus Bijapur, and across relationship types of family members to AGs, where nonliterate households in Bagalkot and pairs with mother or other female members, had more inequitable attitudes than fathers relating to statement 2.

Figure 2(c) and (d) shows the highest proportion of AGs who felt it was not at all important to complete secondary school or have steady employment when they grow up, came from family pairs in which the AG agreed and the family member disagreed to statement 2. In adjusted models (Table 5), AGs in pairs with concordant disagreement to statement 2 (vs. concordant agreement) were more likely to report that completing secondary school was very/somewhat important (vs. not at all important; aOR = 2.35, 95% CI = 1.28–4.32), and that future steady employment was very/somewhat important (vs. not at all important; aOR = 1.77, 95% CI = 1.10–2.86).

**Table 5.** Adjusted Associations Between Statement 2 “There are Times When it is ok for a Women to be Beaten” and Samata Girls’ Baseline Aspirations of Educational Attainment and Future Steady Employment.

	Importance of completing secondary school (very/somewhat vs. not at all)	Importance of future steady employment (very/somewhat vs. not at all)
	Adjusted OR (aOR) (95% CI)	aOR (95% CI)
Level of agreement:		
Both agree	Ref	Ref
AG disagrees but family agrees	1.66 (0.89–3.10)	1.50 (0.89–2.53)
AG agrees but family disagrees	0.86 (0.56–1.33)	0.84 (0.58–1.22)
Both disagree	**2.35 (1.28–4.32)**	**1.77 (1.10–2.86)**

*Note*. All models adjusted for household literacy, household composition (e.g., nuclear family), family member type, household wealth index, family member age, any male sibling, caste/tribe, and district.

Items in **bold** represent statistical significance at *p* < .01.

## Discussion

The results from this study demonstrate that among the SC/ST communities in rural northern Karnataka, AGs/family pairs had high levels of gender attitudes that endorse hegemonic (e.g., dominant/ruling) gender roles and inequitable power dynamics within marriage, and acceptance of VAW. Unsurprisingly, AGs disagreed with statement 1 more than their older generation family members, however, surprisingly within AGs/family pairs, AGs were more likely to believe there are times when it is acceptable for a woman to be beaten compared with family members. We found that AGs who disagreed with their family member's belief that a wife should always obey her husband had greater aspirations for finishing secondary school and having a future career. Increased odds of positive career and education aspirations were only found when both members of the family pairs disagreed with the statement reflecting acceptance of VAW.

To our knowledge, this is the first study to look at similarities and differences in attitudes toward gendered power dynamics between AGs and family members in the Indian context. As such, there is limited comparable evidence. Results from this study found that AGs were more likely to disagree that a wife should always obey her husband, which highlights that positive educational and career aspirations are more likely when AGs are able to form their own gender-equitable attitudes contrary to what attitudes may have been instilled/modeled in the household. However, attitudes centered on the acceptability of VAW was not influenced by family members' attitudes, and the positive association with AGs’ educational and career aspirations was only significant when AGs and their family member both held gender-equitable attitudes. Previous research conducted in Ghana found that educational aspirations mediated the relationships between AGs’ academic self-efficacy and performance (Ansong et al., 2019). Findings further support qualitative evidence from the Samata trial that greater aspirations for the future enabled agency for school attendance and against early marriage (Ramanaik et al., 2020). Thus, the results from this study point to the importance of instilling household-level gender-equitable attitudes through efforts tailored to AGs and their family members independently, as well as efforts to promote agency and voice among AGs.

Although AGs were more likely than family members to disagree that a wife should always obey her husband, they were less likely to disagree with statements surrounding VAW. These results were surprising as we would expect that with improved economic growth and increasing laws and policies surrounding improving gender equality in India, AGs would be less accepting of VAW. Levels of acceptability of VAW among AGs in our study were higher than in a 2014 global study looking at the global prevalence of acceptability and justification of domestic violence, in which 47% of Indian women justified domestic violence (Sardinha & Najera Catalan, 2018). However, these results align with other studies showing that in India, a higher proportion of 15- to 19-year-old's justified ‘wife beating’ compared to any other older age group (Rani & Bonu, 2009). This may be due to internalized gender inequitable attitudes that begin at an early age through gender socialization. Gender socialization is largely influenced by the intergenerational transfer of attitudes around gendered power held within the family unit (Farre & Vella, 2013; Raj et al., 2014). Qualitative research on gender attitudes among adolescents has highlighted how adolescents learn about gender attitudes and roles in the household through direct and indirect messaging by parents and other family members (Kagesten et al., 2016). Also, findings may be indicative of high levels of violence in the home, as previous research has found that children who witness violence or experience violence in the home are more likely to develop attitudes that are accepting of violence (Baron et al., 1988).

High levels of acceptability toward VAW within AG/family pairs are consistent with a 2009 multi-country analysis exploring the prevalence and factors associated with attitudes toward wife beating, in which 57% of Indian women surveyed justified wife beating (Rani & Bonu, 2009). Elevated justification for domestic violence and harassment among a cohort of AGs aged 13 and 14 may result in harmful consequences, such as increased harassment and violence, due to high levels of acceptance of VAW within highly gender inequitable societies (Moonzwe Davis et al., 2014; Rocca et al., 2009). Another explanation could be that the question itself could be constructed as confusing and that girls may not only relate this to domestic violence and perhaps think of women elsewhere who do something criminal, thus believing there has to be some punishment. Moreover, given the age of the participants, AGs in our study may not have had many opportunities to be exposed to supportive environments or personal experiences in which they can begin to form opinions about questioning harmful gender attitudes.

Results from this study are not without limitation. Given the cross-sectional nature of this study, we cannot determine the directionality of effects between gender belief dis/agreement among AG/family member pairs and future aspirations. This study uses cross-sectional data from the baseline survey to examine similarities and differences in gender attitudes between pairs, and how these dis/agreements influence factors that are important mediators to educational success. Given that some evidence suggestions future aspirations may not be predictive of achieving future goals (Frye, 2012), further analysis of the Samata trial data will seek to examine how similarity and differences in agreements across statements regarding gender inequitable attitudes influence trends in school attendance and child marriage over the course of the intervention. Due to the sensitive nature of questions regarding gender attitudes, the results may be subject to differential social desirability bias between AGs and their family members. However, these data were collected prior to the intervention, and thus some of the participants may not have been aware of the preference for gender-equitable attitudes, reducing the susceptibility of social desirability bias. Studying gender attitudes and norms is an evolving field, some preliminary work has identified limitations in asking general statements regarding gender equity as it does not provide granularity in results (Moreau et al., 2019). Thus, future work should pilot more contextual and specific questions around gender attitudes, beliefs, and norms in rural Indian contexts with AGs and their older generational family members.

### Policy and Programming Implications

In a 2001 paper by Mathur et al., the authors argue that to support the reproductive health and well-being of AGs in Nepal, there is a need for research to shift away from risk behaviors to more distal factors influencing behaviors such as societal beliefs, as well as how personal aspirations of younger generations can challenge existing attitudes, beliefs, and societal norms (Mathur et al., 2001). To achieve gender equality and other SDGs such as the provision of quality education (SDG 4), there is a need to shift societal-level gender attitudes, beliefs, and norms that devalue women and girls (Schensul et al., 2015). Results from this study highlight the importance of household and family-level characteristics in the development of gender attitudes, thus strategies to improve and shift gender attitudes may function differently depending on baseline household gender attitudes. Moreover, findings from our study have important implications for the psychological well-being and agency of AGs, as previous research has indicated that hope and future aspirations are often synonymous with better mental well-being (Davids et al., 2016), increased social benefit (Frye, 2012), and agency (Ramanaik et al., 2020). Programming, such as *Girl Rising India*, which uses communication tools and storytelling to inspire social change, has been shown to be effective at shifting harmful attitudes by improving AGs’ voice and agency to stand up to parents and others in their communities against harmful gender attitudes (Vyas et al., 2020). Our data suggest that further efforts are needed to engage with family and the wider community to shift gender attitudes on a large scale. Media exposure promoting positive gender norms, such as serial dramas, is a strategy that may be effective at promoting positive gender attitudes at the household-level (Petraglia et al., 2007).

Although family and community connections are at the heart of Indian society, few studies have implemented family-based interventions to improve education and reduce harmful gender attitudes, despite the high acceptability of these approaches (Guilamo-Ramos et al., 2012; Soletti et al., 2009). While the Samata intervention did include an element that engaged with families, the efforts targeted at family members were unsuccessful in shifting gender norms surrounding girls’ education and child marriage (Prakash et al., 2020). However, results did indicate that secular shifts in increased gender-equitable attitudes indicate that attitudes to girls’ education and marriage are clearly changing. To continue to improve upon and sustain these secular shifts, further research is needed to understand how best to engage families in programming. Results from this study can help to inform future efforts needed to reduce harmful gender attitudes surrounding gender roles and VAW to foster positive aspirations rather than negative behaviors (Mathur et al., 2001), improve equity in education, and support the SRHR of AGs in India.

## Conclusions

These results highlight that AGs hold negative and disparate attitudes about the acceptability of VAW and most attitudes surrounding wives’ duties to obey their husbands are normative within the majority of households. When AGs reported disparate attitudes to family members regarding gender roles in marriage, they held more positive beliefs about their own education and future career, while household-level disagreement to statements regarding the acceptance of VAW was needed to influence positive future educational and career aspirations. As gender socialization is largely influenced by families and begins at an early age, efforts should consider family-level programming that aims to promote positive aspirations and positive modeling of gender-equitable attitudes.

## References

[bibr1-10778012221097142] Ansong D. Eisensmith S. R. Okumu M. Chowa G. A. (2019). The importance of self-efficacy and educational aspirations for academic achievement in resource-limited countries: Evidence from Ghana. Journal of Adolescence, 70(1), 13–23. 10.1016/j.adolescence.2018.11.003 30471622

[bibr2-10778012221097142] Baron L. Straus M. A. Jaffee D. (1988). Legitimate violence, violent attitudes, and rape: A test of the cultural spillover theory. Annals of the New York Academy of Sciences, 528(1), 79–110. 10.1111/j.1749-6632.1988.tb50853.x 3421615

[bibr3-10778012221097142] Basu S. Zuo X. Lou C. Acharya R. Lundgren R. (2017). Learning to be gendered: Gender socialization in early adolescence among urban poor in Delhi, India, and Shanghai, China. Journal of Adolescent Health, 61(4S), S24–S29. 10.1016/j.jadohealth.2017.03.012 28915988

[bibr4-10778012221097142] Beattie T. S. Bhattacharjee P. Isac S. Davey C. Javalkar P. Nair S. Thalinja R. Sudhakar G. Collumbien M. Blanchard J. F. Watts C. Moses S. Heise L. (2015). Supporting adolescent girls to stay in school, reduce child marriage and reduce entry into sex work as HIV risk prevention in north Karnataka, India: Protocol for a cluster randomised controlled trial. BMC Public Health, 15, Article 292. 10.1186/s12889-015-1623-7 25881037 PMC4391662

[bibr5-10778012221097142] Beattie T. S. Javalkar P. Gafos M. Heise L. Moses S. Prakash R. (2019). Secular changes in child marriage and secondary school completion among rural adolescent girls in India. Journal of Global Health Reports, 3, Article e2019041. 10.29392/joghr.3.e2019041

[bibr6-10778012221097142] Bhagavatheeswaran L. Nair S. Stone H. Isac S. Hiremath T. Raghavendra T. Vadde K. Doddamane M. Srikantamurthy H. S. Heise L. Watts C. Schweisfurth M. Bhattacharjee P. Beattie T. S. (2016). The barriers and enablers to education among scheduled caste and scheduled tribe adolescent girls in northern Karnataka, South India: A qualitative study. International Journal of Educational Development, 49, 262–270. 10.1016/j.ijedudev.2016.04.004

[bibr7-10778012221097142] Blanchard A. K. Nair S. G. Bruce S. G. Chaitanya A. T. M. S. Ramanaik S. Thalinja R. Murthy S. Javalkar P. Pillai P. Collumbien M. Heise L. Isac S. Bhattacharjee P. (2018). A community-based qualitative study on the experience and understandings of intimate partner violence and HIV vulnerability from the perspectives of female sex workers and male intimate partners in North Karnataka state, India. BMC Women’s Health, 18(1), Article 66. 10.1186/s12905-018-0554-8 29751752 PMC5948786

[bibr8-10778012221097142] Davids E. L. Roman N. V. Kerchhoff L. J. (2017). Adolescent goals and aspirations in search of psychological well-being: From the perspective of self-determination theory. South African Journal of Psychology, 47(1), 121–132. 10.1177/0081246316653744

[bibr9-10778012221097142] Farre L. Vella F. (2013). The intergenerational transmission of gender role attitudes and its implications for female labour force participation. Economica, 80(318), 219–247. 10.1111/ecca

[bibr10-10778012221097142] Frye M. (2012). Bright futures in Malawi’s new dawn: Educational aspirations as assertions of identity. American Journal of Sociology, 117(6), 1565–1624. 10.1086/664542 PMC3641706 23645932

[bibr11-10778012221097142] García-Moreno, C., Pallitto, C., Devries, K., Stöckl, H., Watts, C., & Abrahams, N. (2013). Global and regional estimates of violence against women: Prevalence and health effects of intimate partner violence and non-partner sexual violence. World Health Organization.

[bibr12-10778012221097142] Government of Karnataka, Bijapur District. (2008). *Human development report. Strengthening state plans for human development (SSPHD) project*. http://www.im4change.org/docs/bijapur_krnkt.pdf

[bibr13-10778012221097142] Guilamo-Ramos V. Soletti A. B. Burnette D. Sharma S. Leavitt S. McCarthy K. (2012). Parent–adolescent communication about sex in rural India: U.S.–India collaboration to prevent adolescent HIV. Qualitative Health Research, 22(6), 788–800. 10.1177/1049732311431943 22232297 PMC3343220

[bibr14-10778012221097142] International Institute for Population Sciences and Macro International. (2007). *National family health survey (NFHS-3), 2005-06: India* (Vol. 1). International Institute for Population Sciences.

[bibr15-10778012221097142] Kagesten A. Gibbs S. Blum R. W. Moreau C. Chandra-Mouli V. Herbert A. Amin A. (2016). Understanding factors that shape gender attitudes in early adolescence globally: A mixed-methods systematic review. PLoS One, 11(6), Article e0157805. 10.1371/journal.pone.0157805 27341206 PMC4920358

[bibr16-10778012221097142] Kumar R. (2007). Dalit literature: A perspective from below. In Ahmad I. Upadhyay S. (Eds.), Dalit assertion in society, history, and literature (pp. 121–136). Orient Blackswan.

[bibr17-10778012221097142] Landry M. Vyas A. Malhotra G. Nagaraj N. (2020). Adolescents’ development of gender equity attitudes in India. International Journal of Adolescence and Youth, 25(1), 94–103. 10.1080/02673843.2019.1590852

[bibr18-10778012221097142] Mathur S. Malhotra A. Mehta M. (2001). Adolescent girls’ life aspirations and reproductive health in Nepal. Reproductive Health Matters, 9(17), 91–100. 10.1016/s0968-8080(01)90012-6 11468851

[bibr19-10778012221097142] Moonzwe Davis L. Schensul S. L. Schensul J. J. Verma R. K. Nastasi B. K. Singh R. (2014). Women’s empowerment and its differential impact on health in low-income communities in Mumbai, India. Global Public Health, 9(5), 481–494. 10.1080/17441692.2014.904919 24766149 PMC4624628

[bibr20-10778012221097142] Moreau C. Li M. De Meyer S. Vu Manh L. Guiella G. Acharya R. Bello B. Maina B. Mmari K. (2019). Measuring gender norms about relationships in early adolescence: Results from the global early adolescent study. SSM – Population Health, 7, Article 100314. 10.1016/j.ssmph.2018.10.014 30581959 PMC6293033

[bibr21-10778012221097142] Office of the Registrar General & Consensus Commissioner. (2011). *Primary census abstract, Karnataka-2011: Data highlights*. https://censuskarnataka.gov.in/Data%20High%20lights-Karnataka-PCA-2011.pdf

[bibr22-10778012221097142] Petraglia J. Galavotti C. Harford N. Pappas-DeLuca K. A. Mooki M. (2007). Applying behavioral science to behavior change communication: The pathways to change tools. Health Promotion Practice, 8(4), 384–393. 10.1177/1524839907301402 17804825

[bibr23-10778012221097142] Philip M. Getachew A. Shaka N. Begum S. (2019). The congruence between adolescents and their parents’ gender-role attitudes in urban slums of Allahabad, India. Gender Issues, 37(3), 223–240. 10.1007/s12147-019-09244-0

[bibr24-10778012221097142] Prakash R. Beattie T. Javalkar P. Bhattacharjee P. Ramanaik S. Thalinja R. Murthy S. Davey C. Blanchard J. Watts C. Collumbien M. Moses S. Heise L. Isac S. (2017). Correlates of school dropout and absenteeism among adolescent girls from marginalized community in north Karnataka, south India. Journal of Adolescence, 61(1), 64–76. 10.1016/j.adolescence.2017.09.007 28968543

[bibr25-10778012221097142] Prakash R. Beattie T. S. Cislaghi B. Bhattacharjee P. Javalkar P. Ramanaik S. Thalinja R. Davey C. Gafos M. Watts C. Collumbien M. Moses S. Isac S. Heise L. (2020). Changes in family-level attitudes and norms and association with secondary school completion and child marriage among adolescent girls: Results from an exploratory study nested within a cluster-randomised controlled trial in India. Prevention Science, 21(8), 1065–1080. 10.1007/s11121-020-01143-1 32720188

[bibr26-10778012221097142] Prakash R. Singh A. Pathak P. K. Parasuraman S. (2011). Early marriage, poor reproductive health status of mother and child well-being in India. Journal of Family Planning and Reproductive Health Care, 37(3), 136–145. 10.1136/jfprhc-2011-0080 21628349

[bibr27-10778012221097142] Raj A. Ghule M. Battala M. Dasgupta A. Ritter J. Nair S. Saggurti N. Silverman J. G. Balaiah D. (2014). Brief report: Parent-adolescent child concordance in social norms related to gender equity in marriage – findings from rural India. Journal of Adolescence, 37(7), 1181–1184. 10.1016/j.adolescence.2014.08.006 25173179 PMC4340658

[bibr28-10778012221097142] Ramanaik S. Collumbien M. Prakash R. Howard-Merrill L. Thalinja R. Javalkar P. Murthy S. Cislaghi B. Beattie T. Isac S. Moses S. Heise L. Bhattacharjee P. (2018). Education, poverty and “purity” in the context of adolescent girls’ secondary school retention and dropout: A qualitative study from Karnataka, southern India. PLoS One, 13(9), Article e0202470. 10.1371/journal.pone.0202470 30183747 PMC6124724

[bibr29-10778012221097142] Ramanaik S. Collumbien M. Pujar A. Howard-Merrill L. Cislaghi B. Prakash R. Javalkar P. Thalinja R. Beattie T. Moses S. Isac S. Gafos M. Bhattacharjee P. Heise L. (2020). ‘I have the confidence to ask’: Thickening agency among adolescent girls in Karnataka, South India. Culture, Health & Sexuality, 24(1), 16–30. 10.1080/13691058.2020.1812118 32969330

[bibr30-10778012221097142] Rani M. Bonu S. (2009). Attitudes toward wife beating: A cross-country study in Asia. Journal of Interpersonal Violence, 24(8), 1371–1397. 10.1177/0886260508322182 18718881

[bibr31-10778012221097142] Rocca C. H. Rathod S. Falle T. Pande R. P. Krishnan S. (2009). Challenging assumptions about women’s empowerment: Social and economic resources and domestic violence among young married women in urban South India. International Journal of Epidemiology, 38(2), 577–585. 10.1093/ije/dyn226 18952621 PMC2734072

[bibr32-10778012221097142] Sardinha L. Najera Catalan H. E. (2018). Attitudes towards domestic violence in 49 low- and middle-income countries: A gendered analysis of prevalence and country-level correlates. PLoS One, 13(10), Article e0206101. 10.1371/journal.pone.0206101 30379891 PMC6209205

[bibr33-10778012221097142] Schensul S. L. Singh R. Schensul J. J. Verma R. K. Burleson J. A. Nastasi B. K. (2015). Community gender norms change as a part of a multilevel approach to sexual health among married women in Mumbai, India. American Journal of Community Psychology, 56(1–2), 57–68. 10.1007/s10464-015-9731-1 26136202 PMC4608233

[bibr34-10778012221097142] Shah P. P. (2011). Girls’ education and discursive spaces for empowerment: Perspectives from rural India. Research in Comparative and International Education, 6(1), 90–106. 10.2304/rcie.2011.6.1.90

[bibr35-10778012221097142] Soletti A. B. Guilamo-Ramos V. Burnette D. Sharma S. Bouris A. (2009). India–US collaboration to prevent adolescent HIV infection: The feasibility of a family-based HIV-prevention intervention for rural Indian youth. Journal of the International AIDS Society, 12(1), Article 35. 10.1186/1758-2652-12-35 19925680 PMC2788348

[bibr36-10778012221097142] StataCorp. (2013). Stata Statistica; Software: Release 13. StataCorp.

[bibr37-10778012221097142] Talboys S. L. Kaur M. VanDerslice J. Gren L. H. Bhattacharya H. Alder S. C. (2017). What is eve teasing? A mixed methods study of sexual harassment of young women in the rural Indian context. SAGE Open, 7(1), 1–10. 10.1177/2158244017697168

[bibr40-10778012221097142] UNICEF. (2018). Annual results report 2017: Education https://www.unicef.org/media/47756/file/Annual_Results_Report_2017_Education.pdf.

[bibr38-10778012221097142] UNICEF. (2020). Empowering adolescent girls and boys in India. Retrieved August 27, 2020, from https://www.unicef.org/india/what-we-do/adolescent-development-participation

[bibr39-10778012221097142] United Nations. (2017). Progress towards the sustainable development goals.

[bibr41-10778012221097142] Vyas A. N. Malhotra G. Nagaraj N. C. Landry M. (2020). Gender attitudes in adolescence: Evaluating the girl rising gender-sensitization program in India. International Journal of Adolescence and Youth, 25(1), 126–139. 10.1080/02673843.2019.1598450

[bibr42-10778012221097142] Zietz S. Das M. (2017). ‘Nobody teases good girls’: A qualitative study on perceptions of sexual harassment among young men in a slum of Mumbai. Global Public Health, 13(9), 1229–1240. 10.1080/17441692.2017.1335337 28580845 PMC6690339

